# Pattern and associated factors of COVID-19 knowledge, attitude and practice (KAP) among COVID-19-comorbid patients: a systematic review and meta-analysis

**DOI:** 10.3389/fpubh.2024.1365744

**Published:** 2024-10-18

**Authors:** Anees ur Rehman, Zermina Tasleem, Sohail Ayaz Muhammad, Muhammad Fawad Rasool, Shahid Shah, Gul Jabeen, Sonia Arif, Lujain Salim Omar Babkair, Yahya Jaber Kadumi, Saleh Alghamdi, Safa S. Almarzoky Abuhussain, Sarah M. Khayyat, Raed Hamed Hilal Alharthi, Mohammad Akbar Hossain, Asma A. Abbas, Mahmoud Essam Elrggal, Abdul Haseeb

**Affiliations:** ^1^Department of Pharmacy Practice, Faculty of Pharmacy, Bahauddin Zakariya University, Multan, Pakistan; ^2^Department of Political Sciences, Bahauddin Zakariya University, Multan, Pakistan; ^3^School of Management Sciences, University Sains Malaysia, Penang, Malaysia; ^4^Department of Pharmacy Practice, Faculty of Pharmaceutical Sciences, Government College University, Faisalabad, Pakistan; ^5^Department of Health Science, Faculty of Public Governance and Business, Mykolas Romeris University, Vilnius, Lithuania; ^6^Faculty of Medicine, University of Oslo, Oslo, Norway; ^7^Infectious Diseases Department, Alnoor Specialist Hospital, Ministry of Health, Makkah, Saudi Arabia; ^8^Pharmacy Department, Alnoor Specialist Hospital, Ministry of Health, Makkah, Saudi Arabia; ^9^Department of Clinical Pharmacy, Faculty of Pharmacy, Al-Baha University, Al-Baha, Saudi Arabia; ^10^Department of Pharmaceutical Practices, College of Pharmacy, Umm Al-Qura University, Makkah, Saudi Arabia; ^11^OPD Pharmacy, Security Forces Hospital, Dammam, Saudi Arabia; ^12^Department of Pharmacology and Toxicology, Faculty of Medicine in Al-Qunfudah, Umm Al-Qura University, Makkah, Saudi Arabia; ^13^Pharmaceutical Care Department, Ministry of National-Guard Health Affairs, Jeddah, Saudi Arabia; ^14^Department of Clinical Pharmacy, College of Health Sciences and Nursing, Al-Rayan Colleges, Al-Madinah Al-Munawarah, Saudi Arabia

**Keywords:** knowledge, attitude, practice, KAP, hypertension, chronic illness, comorbidity, COVID-19

## Abstract

**Background:**

The COVID-19 comorbid population is at higher risk of developing severe health issues like acute respiratory distress syndrome, coagulation syndrome, metabolic acidosis, and septic shock, potentially leading to patient death. Patients’ knowledge, attitudes, and practices (KAP) significantly influence their response to the pandemic and aid in enhancing health policy implementation.

**Objective:**

To identify and evaluate the pattern and associated factors to COVID-19 knowledge, attitude, and practice among individuals with comorbidities.

**Methodology:**

The systematic review followed the PRISMA guidelines. Relevant studies assessing the KAP of comorbid patients were retrieved by carefully searching the PubMed and Google Scholar databases. The appraisal tool for cross-sectional studies was used to determine the quality of the included studies and the risk of biases.

**Results:**

Eighteen studies met the inclusion criteria and were included in the review. The pooled sample size of the included studies was 9,104. Different comorbidities reported in the studies include hypertension, diabetes, psychological disorders, and cancer. Pooled analysis showed that 65% of patients showed good knowledge, 57% of patients showed a positive attitude and 51% of patients followed good practices to manage the COVID-19 in presence of their comorbid condition. Significant factors impacting knowledge, attitude and practice in COVID-19 comorbid patients were ethnicity OR 1.78 [95% CI 1.35–2.32]; educational status 3.2 [2.79–3.58]; urban residence 2.43 [1.65–3.02]; employment Status 1.67[1.34–2.12]; financial Status 4.02[3.66–4.38]; occupation 3.65[3.31–4.25]; information Source 2.64[2.19–3.26]; comorbidity 3.28[2.78–3.61]; and duration of chronic illness 1.59[1.31–2.04].

**Conclusion:**

Comorbid COVID-19 patients showed good knowledge, positive attitude and good practice towards the management of the disease.

## Introduction

The coronavirus disease is an extremely contagious and infectious pulmonary disease, caused by the severe acute respiratory syndrome coronavirus 2 (SARS-COV2). The first outbreak was initiated in Wuhan, China, in December 2019, and soon it blew out throughout the globe due to a higher spread rate (1). It infected a large population of the world in a short time interval. The emergence and widespread prevalence of coronavirus disease have resulted in severe consequences in different dimensions. According to the World Health Organization (WHO), COVID-19 has affected almost 470 million people throughout the globe, with a death rate of 3.4% (2, 3). Due to the high infection rate and associated mortality, COVID-19 was declared as Public Health Emergency of International Concern by the WHO at the start of 2020 (4).

The SARS-CoV-2-infected patients are mostly asymptomatic or generally experience mild symptoms that include fever, dry cough, sore throat, myalgia, fever, and fatigue. Comorbidities may cause different complications in patients suffering from COVID-19. In addition to the above-mentioned symptoms, the comorbid COVID-19 population is more vulnerable to developing acute respiratory distress syndrome, coagulation syndrome, metabolic acidosis, and septic shock, which may result in patient death (5). Thus, COVID-19 patients with associated comorbid conditions, including hypertension, pregnancy, cardiovascular diseases, diabetes, renal disease, and respiratory diseases, are categorized as high-risk patients (6). These high risk comorbid COVID-19 Patients experience a twofold increase in the necessity for mechanical ventilation, and a threefold elevation in the likelihood of developing severe disease or requiring admission to the intensive care unit (ICU) (7, 8). Comorbid COVID-19 patients are at increased risk of mortality as compared to COVID-19 patients without comorbidity. Data collected from several global locations, encompassing both news outlets and scientific publications, has indicated that the overall case-fatality rate of COVID-19, standing at 3.4%, is more than three times greater (7.3%) in people with preexisting co-morbidities (5, 9–11).

Given the lack of a targeted therapeutic intervention for COVID-19, it is imperative that individuals with underlying health conditions adopt additional precautions and rigorously adhere to recommended practices of maintaining physical distance and practising proper hand hygiene. This approach is essential for facilitating the most effective management of their health. The acceptance of these preventive and therapeutic measures is contingent upon an individual’s level of awareness and knowledge regarding the infection caused by COVID-19 (12). KAP surveys are commonly employed in health behavior research to gather data on the knowledge, attitudes, and practices of individuals regarding a specific scientific topic. They exhibit characteristics that are representative of the specific demographic under study. The collected data serves as a means of assessing the state of community education (13). The role of citizens’ knowledge, attitudes, and practices (KAP) has been widely acknowledged as crucial in effectively managing previous pandemics (13). In the context of the 2009 Swine Flu pandemic, health officials were informed by KAP surveys and systematic evaluations about the necessary measures to implement to contain the transmission of emerging viruses and mitigate the impact of future pandemics (14).

The comprehension of individuals’ attitudes helps predict and influence their behavior. Understanding disease states can contribute to improving patient well-being, mitigating disease advancement, and reducing the likelihood of hospitalization and mortality (15, 16). Consequently, it is anticipated that the knowledge, attitudes, and practices (KAP) levels among individuals with comorbidities will serve as the determining factor in their battle against the COVID-19 pandemic and to effectively tailor educational interventions aimed at enhancing the application of health policies (17). This approach is crucial in mitigating the transmission and proliferation of diseases (12).

Given the nature of the COVID-19 pandemic, it is imperative to regularly update scientific literature to facilitate the production of empirical research on interventions and strategies that can enhance the efficacy of efforts aimed at mitigating the impact of the COVID-19 pandemic (18). To achieve this objective, it is imperative to conduct a comprehensive and up-to-date systematic evaluation of the existing body of data. There are a limited number of systematic reviews and meta-analyses available that have examined the knowledge, attitudes, and practises (KAP) related to COVID-19. All the available systematic reviews on KAP of COVID-19 have their own limitations. Few reviews focused on KAP of general population (19–22), some are about KAP of healthcare professionals (20), some are focused on the KAP of specific country (America and Ethiopia) (23, 24). To the best of authors’ knowledge, there has been no comprehensive review conducted to assess the effects of KAP on patients with comorbidities. Taking into consideration the constraints outlined in the existing body of literature, the present systematic review and meta-analysis aimed to identify and evaluate the pattern and associated factors to COVID-19 knowledge, attitude, and practice among individuals with comorbidities. This study has the potential to inform the formulation of policies aimed at providing support to vulnerable groups, particularly individuals with comorbidities, to mitigate mortality rates, minimise the adverse effects on the healthcare system, and prevent economic downturn.

## Methodology

### Search process

The systematic review followed the PRISMA guidelines (25). Relevant studies assessing the KAP of comorbid patients were retrieved by carefully searching the PubMed and Google Scholar databases until September 15, 2023 (the date last searched). For PUBMED Search terms used were, “COVID-19” OR “SARS-CoV-2” OR “2019 nCoV” OR “novel coronavirus” OR “new coronavirus” OR “severe acute respiratory syndrome coronavirus 2” OR “Wuhan and coronavirus” OR “pathogenesis” OR “infection” OR “transmission” AND “KAP” OR “knowledge” OR “attitude” OR “perception” OR “practice” OR “awareness” OR “action” AND “comorbidity” OR “diabetes” OR “hypertension” OR “pulmonary disorder” OR “renal failure” OR “cancer” OR “chronic disorder.” Moreover, included reference lists were manually searched to address all the published evidence.

### Eligibility criteria

The systematic review included all the studies meeting the set inclusion criteria (1) cross-sectional studies, (2) well defined COVID-19 patients, (3) reporting at least one comorbid condition (diabetes, hypertension, pulmonary disorder, renal failure, and cancer), (4) investigating at least one or all components of KAP (knowledge, attitude and practice) model in COVID-19 patients, (5) published in peer reviewed journal. KAP studies in the general population, healthcare workers, systematic reviews, and any study published in a language other than English were excluded. The results measured in the included studies were knowledge, attitudes, and practices related to COVID-19 in comorbid patients.

The study selection approach employed the explicit method to prevent biases (26, 27). Explicit method involves rigorously adhering to stated inclusion criteria during the search process. The objective is to offer more dependable conclusions. The papers that met the inclusion and exclusion criteria, as determined by the abstract and title, were carefully examined by three reviewers. Any disagreements between the reviewers were resolved through discussion until a consensus was achieved.

### Selection of studies and data extraction

Titles and abstracts were screened to assess articles for inclusion in the study. Two reviewers independently extracted data in a predesigned data extraction sheet in Excel software. Extracted information included author name, country, year, study period, study design, inclusion and exclusion criteria, method, target population, number and age of participants, study outcomes (knowledge, attitude, practice) and their associated factors. Any discrepancies between the two reviewers were resolved through discussion or by the involvement of the third reviewer (principal investigator) unless consensus was reached.

### Study outcomes

The primary outcomes of the meta-analysis focused on three key areas: knowledge (including understanding of symptoms, transmission methods, high risk populations, incubation and isolation periods, virus fatality rates, and prevention and treatment of COVID-19 infection), attitude (regarding the control and management of COVID-19), and practice (such as hand hygiene, social distancing, using face masks, avoiding crowded places, and adhering to isolation measures). The included studies collected responses using “Yes/No/Do not know” or “Strongly agree/Agree/Neutral/Disagree/Strongly disagree” items. The responses of “Yes,” “True,” or “Strongly agree/Agree” were considered positive responses for the analysis. The secondary outcomes encompassed the reported factors that affect the knowledge, attitude, and practice of comorbid COVID-19 patients towards the management of disease.

### Quality assessment and risk of biasness

The appraisal tool for cross-sectional studies (AXIS) was used to determine the quality of the included studies and the risk of biases (28). The tool has 20 questions, and each question carries one score. Based on the quality assessment tool scores, the studies were categorized as follows: scores (>15) were considered to have less biasness and good quality, (10–15) were considered to have moderate biasness and fair quality while (<10) were considered to have higher biasness and poor quality.

### Statistical analysis

The retrieved studies provided quantitative data on the percentage of knowledge, attitudes, and practices (KAP) related to COVID-19 among patients with comorbidities. The pooled proportion of COVID-19 knowledge, attitudes, and practises (KAP) was computed and displayed using forest plots. The data on KAP-related factors was presented in the form of odds ratios (95% CI) for each component. The analysis was performed using a random effects model. The I^2^ index was utilized to examine the heterogeneity of the study. Values of I^2^ ≥ 50% show higher heterogeneity. Sensitivity tests were conducted to assess the impact of the quality score categories of the included studies (results not shown). No missing data were reported in the study. A significance level of *p* < 0.05 was deemed as statistically significant. Statistical software package Stata V.16 (StataCorp, United States) was used for statistical analyses.

## Results

### Search results

**Figure 1:**
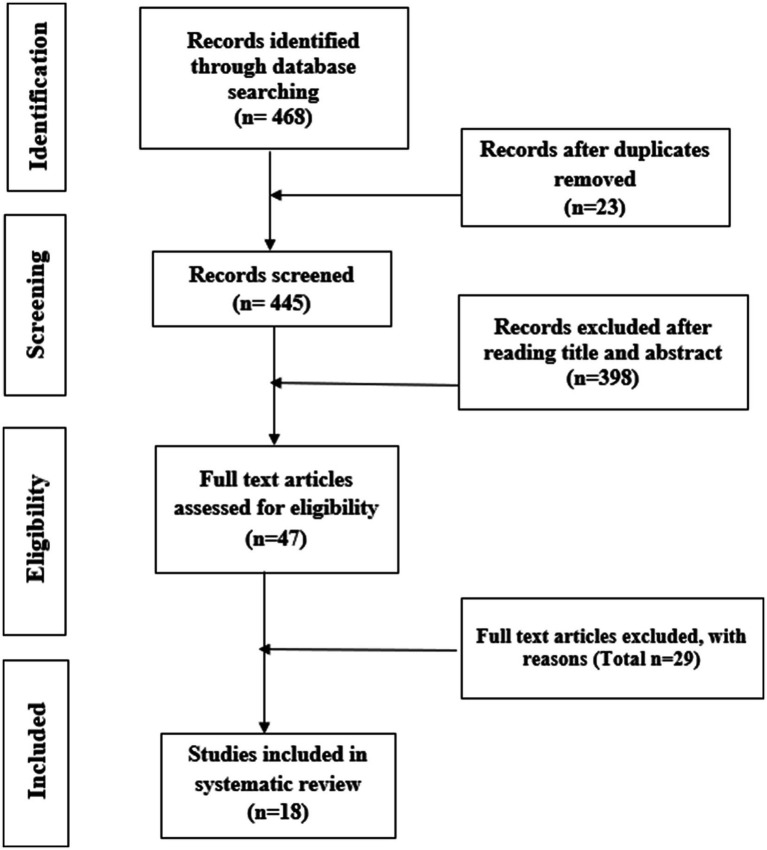
Systematic study selection process, in line with the PRISMA guidelines (25).

Originally, 468 articles were obtained after the initial search. After removing 23 duplicates, the remaining 445 articles were screened by title and abstract to retain 47 articles for full-text review. Eighteen studies met the inclusion criteria and were included in the review. The detailed inclusion and exclusion process is shown in Figure 1.

### Characteristics of selected studies

Among the included studies, seven were performed in Ethiopia, two each in India, Pakistan, and Iran, and one each in Kuwait, Saudi Arabia, the US, Rwanda, and Romania. All the studies were cross-sectional. The pooled sample size of the included studies was 9,104, ranging from 64 to 1,585. The ages of the included patients range from 18 to 80 years. Different comorbidities reported in the studies were hypertension, diabetes type 1 and 2, thyroid disorders, psychological disorders, depression, cancer, chronic pulmonary disease, heart disease, kidney disease, and HIV/AIDS. Eight studies show a low risk of bias, whereas 10 studies show a moderate risk of bias. The summaries of the included studies are demonstrated in Table 1.

**Table 1:** Summaries of the included studies.

Reference	Country	Year	Study design	Questionnaire	Participants	Age	Chronic Condition	Knowledge	Attitude	Practice	Factors Associated with KAP	Risk of Biasness
Taye et al. (29)	Ethiopia	2020	Cross-sectional	Self-developed	423	44.6	Hypertension, Diabetes	Good =37.59%	Positive =79.2%	Good =10.4%	Age, Occupation, ethnicity, source of information	Moderate
Addis SG et al. (13)	Ethiopia	2021	cross-sectional	Pre-tested,	413	≥18	NA	Good =34.6% Moderate =35.1% Poor =25.7%	Positive =81.4% Moderate =12.1% Negative = 6.5%	Good =40.7% Moderate =24.7% Poor = 34.6%	age, urban residency, secondary education, comorbidity, gender	Low
Akalu et al. (18)	Ethiopia	2022	cross-sectional	Pre-tested, structured	404	≥18	Hypertension, diabetes mellitus, heart disease, chronic lung disease	Poor =33.9%	Negative =41%	Poor =47.3%	Age, educational status, urban residence, financial status	Moderate
Iyasu et al. (30)	Ethiopia	2021	Cross-sectional	Self-developed	422	NA	NA	Poor = 35.1%.	Negative =40.5%	Poor =48.8%	Age, Education, Urban residency	Moderate
Geleta et al. (31)	Ethiopia	2022	Cross-sectional	structured questionnaire	360	NA	Hypertension	Good =58.3%	Positive =55.3%	Good =58.3%	NA	Moderate
Legese et al.(32)	Ethiopia	2023	cross-sectional study	WHO guidelines	319	≥18	NA	Good =51.1%	Positive =59.9%	Good =49.2%	Age, gender, level of education, religion, marital status, occupation, urban residence	Low
Adella et al. (33)	Ethiopia	2022	cross-sectional	structured, interviewer-administered	409	≥18	Hypertension, Diabetes mellitus	Good =79.2%	Positive =70.9%	Good =58.2%	Age, education level	Low
Saeed et al. (12)	India	202`	cross-sectional online survey	Self-designed	260	≥30	Hypertension, Diabetes mellitus, Thyroid disorders	Good =82.7%	Positive =75.0%	Good =51.2%	Age ≥ 50, Comorbidity, Education, occupation	Moderate
Pal et al. (16)	India	2020	Cross-sectional	Self-developed	223	18–30	Type 1 diabetes mellitus (T1DM)	Good =83%	Positive =98%	Good =64%	Age, financial status, duration of disease, education, urban residence	Low
Rajan et al. (34)	Bangladesh & Pakistan	2022	Cross-sectional	Self-developed,	845 & 454	N	Schizophrenia, bipolar disorder, schizoaffective disorder, depression	Bng Good =74.61% Pak Good =60%	Bng Negative =29.94% Pak Negative =44.96	Bng Poor =26.5% Pak Poor =28.4%	Gender, information source	Moderate
Khatta et al. (35)	Pakistan	2022	cross-sectional	structured questionnaire	208	≥18	breast cancer, skin cancer, lung cancer	Good =93%	Positive =94.2%	Good =90%	Gender, marital status, employment status,	Moderate
Alsaleh et al. (36)	Kuwait	2023	cross-sectional	self-administered	251	≥18	type 1 or type 2 diabetes	Moderate =71.1%	Positive =90.9%	Good =83.6%	Age, gender, marital status, financial status	Moderate
Subyani et al. (37)	Saudia Arabia	2022	cross-sectional	NA	64	18–65	Type 1 Diabetes	Good = 6.25% Moderate =43.75% Poor =11.50%	Positive =42.2% Negative =37.5	Good =64.1% Poor =48.8%		Moderate
Wolf et al. (8)	US	2020	Cross-sectional	Interviews	630	23–88	Heart disease, pulmonary disease, diabetes, hypertension, polypharmacy	Poor =28.3%	Negative =30.2%	Poor =21.9%	Ethnicity, Financial status, Education	Low
Mohamadian et al. (38)	Iran	2023	cross-sectional	Self-developed	368	≥18	Type2 Diabetes	Good =74.22%	Positive =72.88%	Good =70.51%		Low
Jasim Alsadaji et al. (39)	Iran & Iraq	2021	Cross-sectional	Self-developed	1,000	NA	Respiratory disease, heart disease, hypertension, Kidney disease, Diabetes	Good 49.33%	Positive =73.30	Good =74.62%	teaching hospitals	Moderate
Iradukunda et al. (40)	Rwanda	2021	cross-sectional	WHO recommendations	376	NA	HIV/AIDS	Good =97%	Positive =74%	Good =90%	Age, gender, urban residence, employment, duration of chronic illness	Low
Gheorghe et al. (17)	Romania	2021	Cross-sectional	WHO recommendation	1,585	17–87	Hypertension, Diabetes, Cardiac Diseases, Cancer	Good =10.8% Moderate =44.3%	Positive =32.6%	Good =55.5%		Low

KAP, Knowledge, attitude and practice; Bng: Bangladesh; Pak: Pakistan.

### The pattern of knowledge towards COVID-19

**Figure 2:**
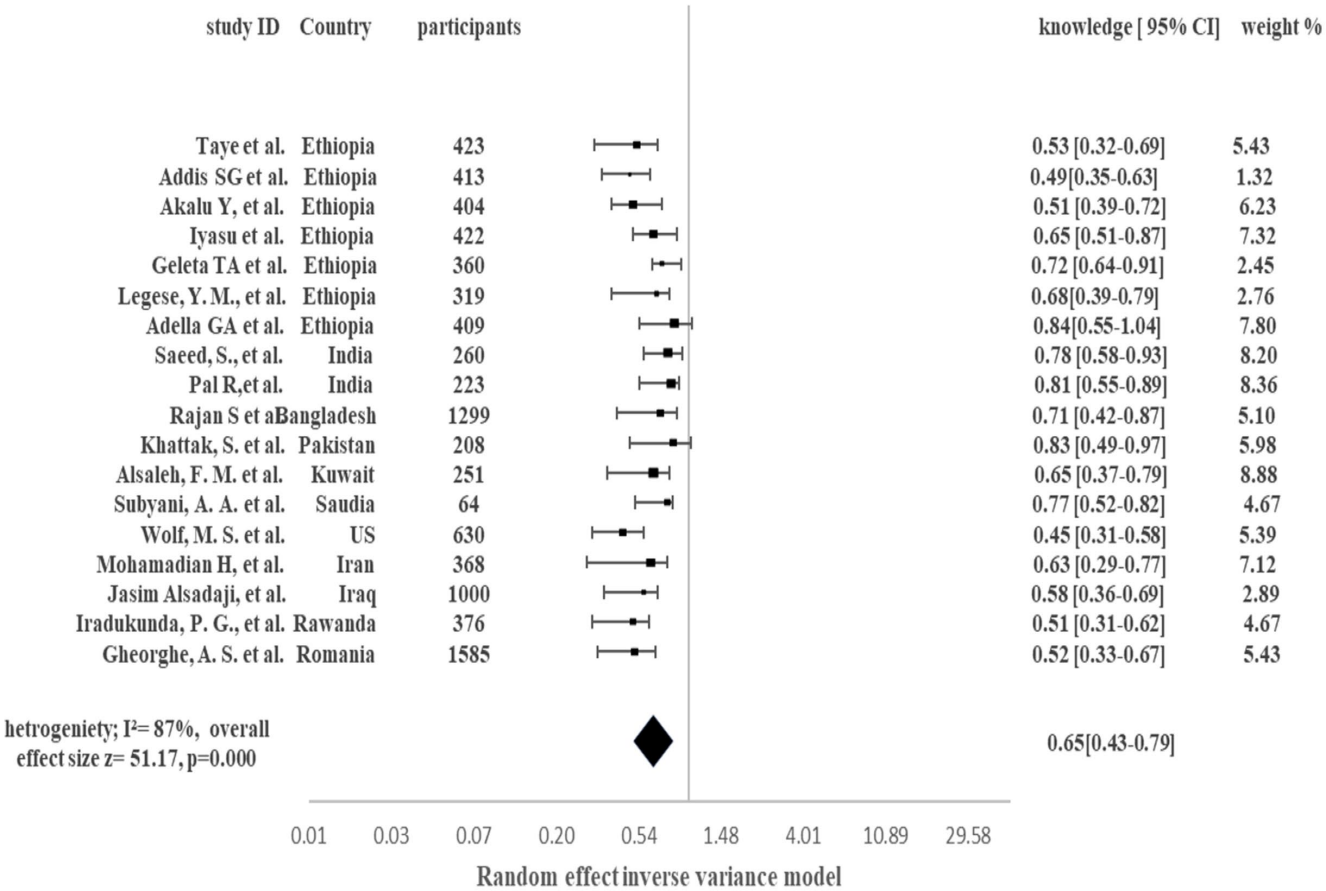
Pooled proportion of Knowledge in Comorbid COVID-19 patients.

Figure 2 demonstrates the pooled proportion of the comorbid population with good knowledge about COVID-19 in the presence of their comorbid condition. Overall, 65% of the comorbid patients acquired good knowledge about COVID-19 and associated comorbidity (95% CI 43–79%, *p* < 0.001, I^2^ = 87%). Pooled analysis showed that in Ethiopia the comorbid population with knowledge was 49 to 84%. Country-wise, India 81% [95% CI 55–89%], Bangladesh 71% [95% CI 42–87%], Pakistan 83% [95% CI 49–97%], Kuwait 65% [95% CI 37–79%], Saudia Arabia 77% [95% CI 52–82%], US 45% [95% CI 31–58%], Iran 63% [95% CI 29–77%], Iraq 58% [95% CI 36–69%], Rwanda 51% [95% CI 31–62%], Romania 52% [95% CI 33–67%] population acquire good knowledge regarding management of COVID-19 along with comorbid condition. High Heterogenicity (I^2^ = 87%) was reported among studies.

### The pattern of attitude towards COVID-19

**Figure 3:**
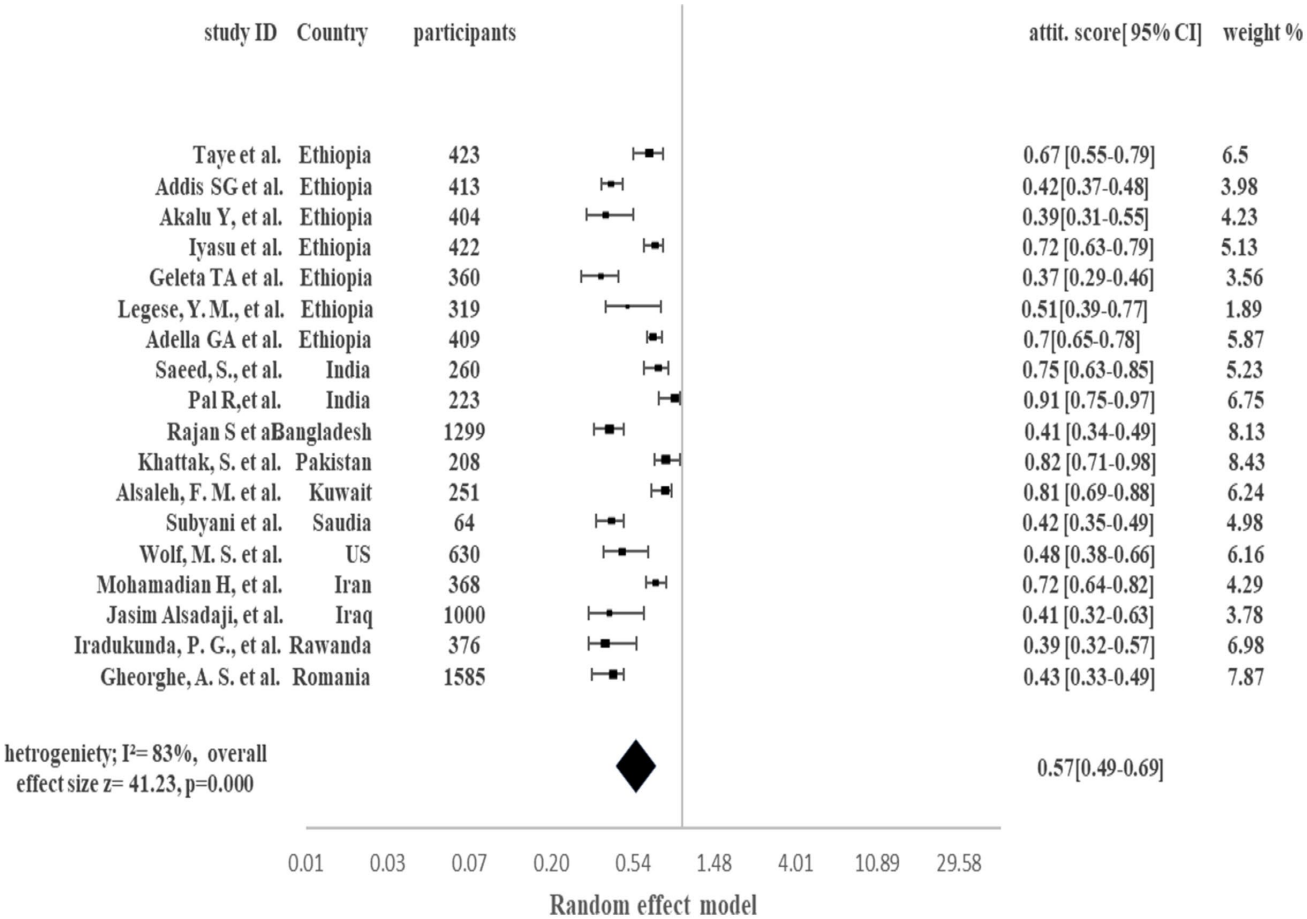
Pooled proportion of positive attitude in Comorbid COVID-19 patients.

Figure 3 demonstrates the pooled proportion of the comorbid population with a positive attitude toward COVID-19 in the presence of their comorbid condition. Overall, 57% of the comorbid patients showed a positive attitude to managing COVID-19 and associated comorbidity (95% CI 49–69%, *p* < 0.0001, I^2^ = 83%). Pooled analysis showed that in Ethiopia the comorbid population with a positive attitude to manage COVID-19 and associated comorbidity were 37 to 72%. Country-wise, India 91% [95% CI 75–97%], Bangladesh 41% [95% CI 34–49%], Pakistan 82% [95% CI 71–98%], Kuwait 81% [95% CI 69–88%], Saudia Arabia 42% [95% CI 35–49%], US 48% [95% CI 38–66%], Iran 72% [95% CI 64–82%], Iraq 41% [95% CI 32–63%], Rwanda 39% [95% CI 32–57%] and Romania 43% [95% CI 33–49%] population showed positive attitude to manage COVID-19 along with comorbid condition. High Heterogenicity (I^2^ = 83%) was reported among studies.

### The pattern of practice towards COVID-19

**Figure 4:**
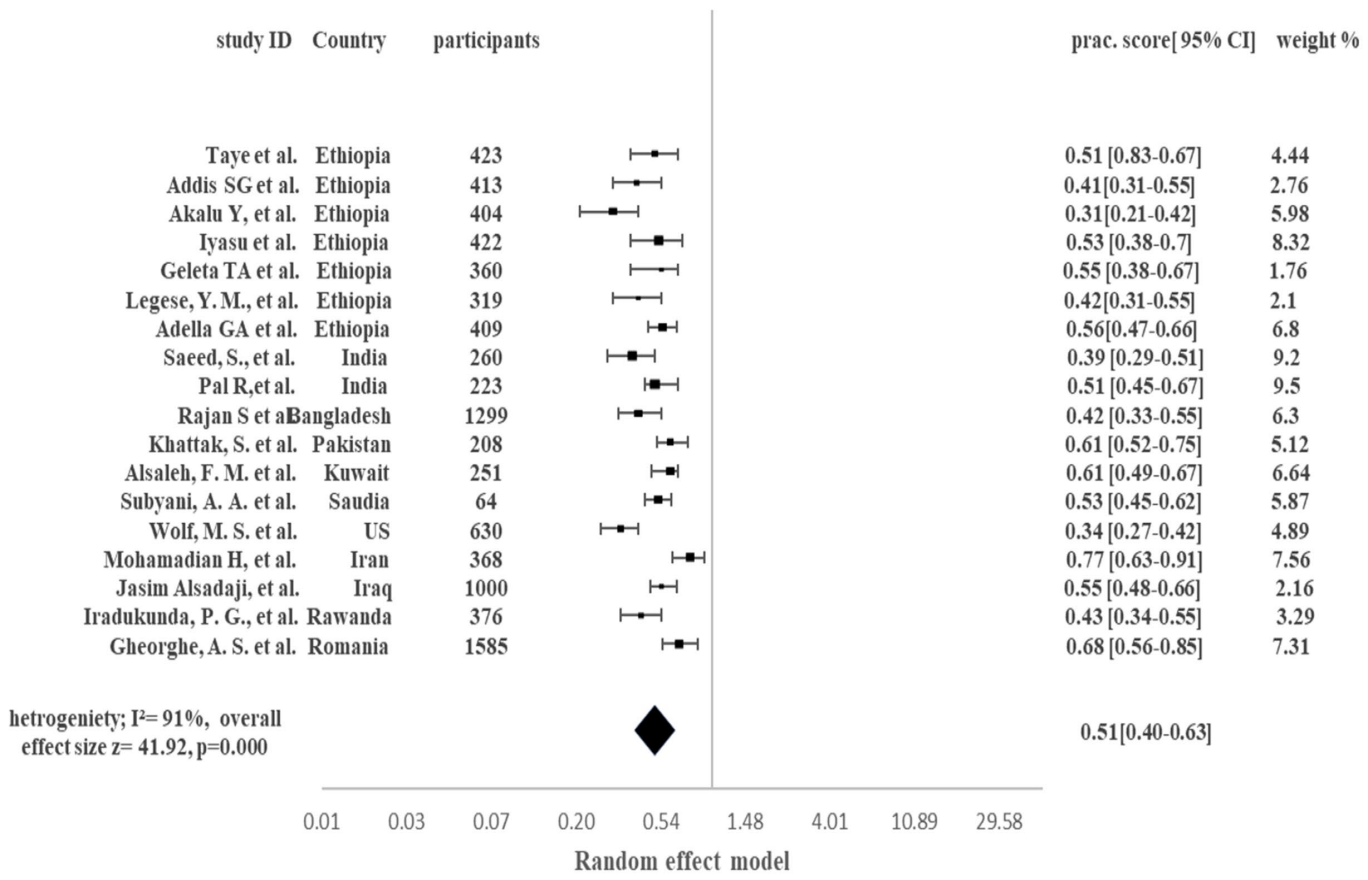
Pooled proportion of good preventive practices in Comorbid COVID-19 patients.

Figure 4 demonstrates the pooled proportion of the comorbid COVID-19 population following good preventive practices to manage the COVID-19 in presence of their comorbid condition. Overall, 51% of the comorbid COVID-19 patients adopted good preventive practices to manage the COVID-19 in presence of their comorbid condition (95% CI 40–63%, *p* < 0.0001, I^2^ = 91%). Pooled analysis showed that in Ethiopia 31 to 56% of patients adopted good preventive practices. Country-wise, India 51% [95% CI 45–67%], Bangladesh 42% [95% CI 33–55%], Pakistan 61% [95% CI 52–75%], Kuwait 61% [95% CI 49–67%], Saudia Arabia 53% [95% CI 45–62%], US 34% [95% CI 27–42%], Iran 77% [95% CI 63–91%], Iraq 55% [95% CI 48–66%], Rwanda 43% [95% CI 34–55%] and Romania 68% [95% CI 56–85%] population were following good preventive practices to manage the COVID-19 in presence of their comorbid condition. Higher heterogenicity (I^2^ = 91%) was reported among studies.

### Factors related to COVID-19 knowledge attitude and practice

**Figure 5:**
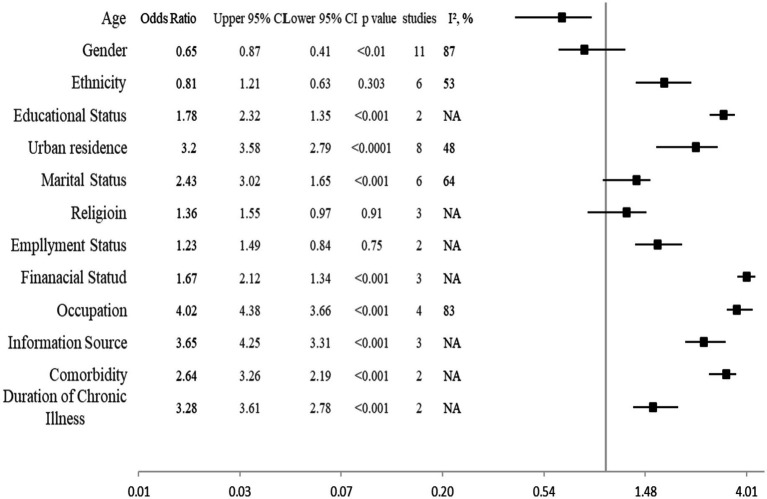
Factors affecting the knowledge, attitude, and preventive practices to manage the COVID-19 in presence of comorbidity.

Different factors affecting the knowledge, attitude, and preventive practices to manage the COVID-19 in presence of comorbidity are shown in Figure 5 with odd ratios, 95% CI, *p* values, I^2^ statistics and forest plot. Significant factors (OR [95% CI]) impacting knowledge, attitude and practice in COVID-19 comorbid patients were ethnicity 1.78 [1.35–2.32]; educational status 3.2 [2.79–3.58]; urban residence 2.43 [1.65–3.02]; employment Status 1.67[1.34–2.12]; financial Status 4.02[3.66–4.38]; occupation 3.65[3.31–4.25]; information Source 2.64[2.19–3.26]; comorbidity 3.28[2.78–3.61]; and duration of chronic illness 1.59[1.31–2.04].

## Discussion

We recognized a limited body of evidence determining the pattern and associated factors of COVID-19 knowledge, attitude, and practice among comorbid COVID-19 patients. Our results showed good knowledge, a positive attitude and good practice to manage COVID-19 in the presence of comorbidity. In addition, good knowledge, positive attitude and good practice were related to age, ethnicity, educational status, urban residence, employment status, financial status, occupation, information Source, comorbidity and duration of chronic illness. Age was reported as an important factor in 11 studies, educational status in eight studies, urban residence and gender in six studies, financial status in four studies, employment status, marital status and occupation in three studies and ethnicity, religion, information source, comorbidity and duration of chronic illness in two studies each.

Adequate knowledge and awareness of preventive strategies are important to manage COVID-19 in comorbid patients (41). Our results showed good knowledge about COVID-19 in 65% of the COVID-19 comorbid patients. Most of the patients were aware of the symptoms of COVID-19 including dyspnea, sore throat, fever, chills, and associated risks due to the comorbid conditions. Cancer patients and diabetes patients’ cardiovascular patients were able to distinguish the COVID-19 symptoms and their respective disease symptoms. Our results are in line with the previous research that shows higher knowledge about COVID-19 management in comorbid patients (35, 36). This may be due to the fact chronic disease patients are in regular contact with healthcare professionals due to their disease condition. Frequent contact with healthcare providers and discussions may improve their knowledge of the pandemic COVID-19.

In our results, 57% of the comorbid patients showed a positive attitude to managing COVID-19 and associated comorbidity. Most of the patients knew COVID-19 may cause more harmful effects in the presence of their disease. Cancer patients believe that COVID-19 increases the risk of mortality so they must adopt extra preventive measures to avoid COVID-19 exposure. Diabetic patients think that they need to manage their diet and physical activity to improve immunity against COVID-19. Our results are in line with the previous literature which shows a positive attitude of 58 and 47% of hypertension and diabetic patients, respectively, toward COVID-19 management (31, 32). Usually, chronic disease patients have better disease management knowledge as compared to healthy people (42–44). So, most of them believe that government policies and restrictions to prevent COVID-19 are for the benefit of the people and can control the pandemic.

Overall, published articles showed the constructive attitude of the participants about COVID-19, which is consistent with the findings of the study conducted in Indonesia (96%) (45). It could be due to better health policies and knowledge about the disease. However, the study done in Northern Ethiopia revealed that 40.5% of the included participants showed a negative attitude towards the prevention of COVID-19 (30). The possible reason for negative attitudes can be poor knowledge about the disease and the unfamiliar nature of COVID-19. Our finding demonstrated that a substantial part of the comorbid patients were concerned about the probability of COVID-19 infection and mortality due to infection. These findings were consistent with those in the Philippines and the United States (8, 46).

Regarding practice, in most studies, participants adopted regular measures to prevent COVID-19 disease. In pooled analysis, 57% of comorbid patients adopted preventive measures to avoid COVID-19. Diabetic patients believe regular physical activity is necessary for them to manage diabetes but they must avoid parks and crowded places to avoid COVID-19 exposure. Comorbid patients need to visit the pharmacy regularly to refill prescriptions or take insulin. Most of the comorbid patients left their houses to visit pharmacies. Policies to provide insulin and other medicines at doorsteps can reduce pharmacy visits and further improve preventive practices in comorbid patients.

This systematic review revealed several factors associated with KAP towards COVID-19 among participants including age, ethnicity, educational status, urban residence, employment status, financial status, occupation, information Source, comorbidity and duration of chronic illness. Younger age was associated with better KAP toward COVID-19 than older age. Different studies reported that people younger than 40 years had better knowledge and positive attitudes toward the management of their disease (16). This may be because young people are more frequent users of social media and adopt change easily. Social media is a key source of information these days. So, this may be the reason for better COVID-19-related KAP in young people. Occupation, financial status and education were also reported as significant factors to impact KAP in comorbid COVID-19 patients in previous research. Studies conducted in Ethiopia, India and Egypt reported occupation, financial status and education as contributing factors to the impact of KAP in comorbid COVID-19 patients (12, 30, 47). Comorbidity and duration of chronic illness were also associated with better KAP in comorbid COVID-19 patients. With an increase in the duration of disease self-management practice and knowledge improve. Research shows that compliance increases with the duration of chronic disease. Thus, with the increased duration of the disease, people may become more conscious of the management of a disease.

Higher heterogeneity was reported among COVID-19 KAP studies. The higher heterogeneity may be due to due to variations in illness manifestation, characteristics of the population being studied, and the methodologies employed in the research (48, 49). The diverse demographics of these research participants, regional variations in healthcare systems, environmental variables, population genetics demographic factors, comorbidities, and the varying severity of COVID-19 lead to heterogeneity. Diagnostic and testing methods are also a factor in the diversity. Heterogeneity can also be caused by variations in study design and methodologies (26, 27). The quality of data and the outcomes obtained differ depending on the type of study conducted, such as observational studies, randomised controlled trials, retrospective studies, or prospective studies. The variety of findings is influenced by the variations in data collection procedures, duration of follow-up, and outcome measures. Study outcomes are also influenced by epidemiological factors such as pandemic waves and new variants that have varying levels of transmissibility and pathogenicity (22, 38, 42).

Notwithstanding its robustness, this study has few limitations. Most of the research conducted took place during the epidemic and primarily consisted of online or telephonic questionnaires. Consequently, individuals lacking access to these amenities may have been deprived of participation. The presence of diverse methods resulted in increased heterogeneity in the studies. Furthermore, the exclusion of grey literature, which was not included in this study due to the availability of sufficient peer-reviewed data, may potentially lead to the omission of certain information. Despite a few limitations, this study has several strengths. The study identified a significant portion of the accessible data and yielded valuable insights into the Knowledge, Attitudes, and Practices (KAP) regarding COVID-19 among individuals with comorbidities. To the best of the author’s knowledge, this study was the first systematic review and meta-analysis to evaluate knowledge attitudes and practices towards COVID-19 and its associated factors in comorbid patients. The anticipated outcomes of the study are expected to bring attention to areas of research that have not been well addressed. This information will be valuable for health professionals in making informed decisions and for developing policies and programs that are specifically targeted at addressing these gaps. The findings have the potential to enhance our understanding of the impact of the pandemic on individuals with chronic diseases, in terms of their knowledge, attitudes, and practices (KAP). Consequently, this could potentially contribute to the enhancement of patient safety and the provision of high-quality care, while also mitigating the transmission of COVID-19 among individuals at a heightened risk.

The COVID-19 pandemic has highlighted the importance of prioritizing vulnerable populations, particularly those with chronic conditions. Millions have died due to inadequate preparedness and responses. In addition to the implementation of efficacious solutions for the prevention and treatment of disease, a positive attitude and practice of the population to adopt preventive measures is also important. Our research shows that by giving priority to vulnerable groups, such as individuals with other chronic illnesses, it is possible to implement focused interventions, enhance access to crucial healthcare services, and implement public health policies (50). Implementation of effective strategies to improve knowledge, including information sources, targeted information, and enhanced telecommunication contact with healthcare providers can improve the attitude of comorbid patients toward the pandemic control. Moreover, media partnerships, digital educational materials, online medicine refills, extended medical supply distribution, hotlines, health protocols, and home visits can be effective in protecting individuals and maintaining care.

## Data availability statement

The raw data supporting the conclusions of this article will be made available by the authors, without undue reservation.

## Author contributions

AR: Conceptualization, Formal analysis, Supervision, Writing – original draft. ZT: Supervision, Writing – review & editing. SM: Software, Writing – review & editing. MFR: Writing – review & editing. SS: Writing – review & editing. GJ: Formal analysis, Writing – review & editing. SA: Formal analysis, Writing – review & editing. LO: Writing – review & editing. YK: Writing – review & editing. SaA: Writing – review & editing. SSA: Writing – review & editing. SK: Writing – review & editing. RA: Writing – review & editing. MH: Writing – review & editing. AA: Writing – review & editing. ME: Writing – review & editing, Resources. AH: Writing – review & editing.
